# The Possibilities and Importance of Assessing the Left Atrioventricular Coupling Index Using Various Diagnostic Imaging Methods in an Adult Population: A Comprehensive Review

**DOI:** 10.3390/jcdd12040110

**Published:** 2025-03-22

**Authors:** Małgorzata Poręba, Krzysztof Kraik, Igor Zasoński, Oskar Ratajczyk, Łukasz Paździerz, Angelika Chachaj, Rafał Poręba, Paweł Gać

**Affiliations:** 1 Department of Biological Principles of Physical Activity, Wroclaw University of Health and Sport Sciences, 51-612 Wrocław, Poland; 2 Students Scientific Association of Cardiovascular Diseases Prevention, Wroclaw Medical University, 50-556 Wrocław, Poland; 3 Department of Angiology, Hypertension and Diabetology, Wroclaw Medical University, 50-556 Wrocław, Poland; 4 Department of Environmental Health, Occupational Medicine and Epidemiology, Wroclaw Medical University, 50-345 Wrocław, Poland

**Keywords:** left atrioventricular coupling index, heart failure, atrial fibrillation, echocardiography, cardiac magnetic resonance

## Abstract

Cardiovascular diseases are a leading cause of death worldwide, and they are becoming even more frequent due to the aging of society. Due to this fact, new parameters that are useful in diagnosing, as well as in assessing, the risk of cardiovascular events, and in future prognosis estimation, should be developed. The left atrioventricular coupling index (LACI) has been recently introduced as the one parameter meeting these criteria. The current review aims to collect all available data and assess whether the LACI may be a valuable tool in daily clinical practice, and, simultaneously, to direct future research on the subject. The LACI is a parameter that can be calculated based on echocardiographic, cardiac CT and CMR examinations. It appears to be of use in several cardiovascular diseases, especially heart failure and atrial fibrillation, both in diagnostics and as a prognostic marker. Moreover, the LACI is a useful marker in cardiomyopathies, myocardial infarction, beta-thalassemia major and light-chain amyloidosis. However, the number of studies on the subject of LACI is limited, and some of these studies are based on the same cohort of patients. Future studies should take up the subject of the LACI, especially when it comes to the value of calculating the LACI based on various imaging techniques, including echocardiography.

## 1. Introduction

According to the World Health Organization (WHO), cardiovascular diseases (CVDs) are the main cause of death; that is, they are responsible for around 32% of all deaths around the world [1]. Although methods of diagnosis and treatment have improved, recently, CVD has become even more frequent, and has grown into the greatest problem faced by health systems worldwide. The high prevalence of CVD is explained by the unhealthy lifestyle in our societies, which includes mainly a lack of physical activity and an improper diet rich in monosaccharides and saturated fat, as well as a smoking habit, and the complexity of the problem is still under debate and extensive research. The main approach to reducing CVD prevalence is primary prevention, which relies on implementing guidelines in this field that promote a healthy lifestyle. Most cardiovascular disorders are chronic diseases with symptoms becoming aggravated with time, often leading to acute complications, and eventually to sudden cardiac death (SCD). Therefore, it is important to recognize them early, so that treatment can be implemented before advanced stages develop. Early interventions help to prevent or slow down the deterioration of the patient’s disease and quality of life. Laboratory tests, electrocardiography (ECG) and cardiac imaging methods are used in the diagnostic process of cardiovascular diseases, in addition to physical examination. Currently, the diagnostic evaluation of cardiovascular diseases is based on invasive and non-invasive methods, where the latter includes imaging techniques such as transthoracic and transesophageal echocardiography (TTE and TEE), computed tomography (CT) of the heart and cardiac magnetic resonance (CMR).

Cardiac imaging techniques can be used to assess several parameters frequently used to evaluate the function of the ventricles and the atria. These parameters include the left ventricular ejection fraction (LVEF), left ventricular end-systolic volume (LVESV), left ventricular end-diastolic volume (LVEDV), global longitudinal strain of left ventricle (LV GLS), left ventricle mass index (LVMI) and left atrial volume index (LAVI). The LVEF reflects the myocardial performance of the left ventricle, and is used to determine the severity of heart failure [2]. The LVEF is calculated by multiplying the ratio of stroke volume to LVEDV by 100%, and its normal value is 50–70%. However, this parameter cannot be used for the prognosis of heart failure with preserved ejection fraction (HFpEF) and with mildly reduced ejection fraction (HFmrEF). GLS describes the deformation of the myocardium in the longitudinal axis during the cardiac cycle. Its normal values range from 18% to 20%. GLS is particularly helpful in the prognosis of patients with HF with LVEF > 35%. Furthermore, the LAVI (left atrial volume index) is calculated as the volume of the left atrium (LA) divided by the body surface area (BSA), and it is useful in the prediction of mortality in CAD and after acute myocardial infarction (AMI) [3]. The LAVI can also be used to predict hospitalization due to HF.

In addition to well-known parameters used in everyday clinical practice, researchers are searching for new parameters that could be applied in the diagnostics and prognostics of cardiovascular diseases. The limitation of most parameters is that they assess the function of either the left ventricle or left atrium, rather than assessing both chambers as a whole, and therefore, it is necessary to develop parameters that can represent the interactions between these chambers of the heart. One such parameter is the left atrioventricular coupling index (LACI), which is the subject of the current review of the scientific literature. The aim of the current review is to summarize up-to-date knowledge about the LACI, to find gaps and inconsistencies in the current state of knowledge and propose a direction for further research dealing with the subject of the LACI.

## 2. Methods

### 2.1. Search Strategy

The review was conducted by searching the Embase, PubMed and Cochrane Library databases. The search process took place between December 2024 and January 2025, and included studies published before this date, meeting the topic of the article and its criteria. The following formula was used during the search in Embase: ‘atrioventricular coupling index’ OR (atrioventricular AND (‘coupling’/exp OR coupling) AND (‘index’/exp OR index)). The keywords used during the search for articles in the PubMed and Cochrane Library databases were ‘atrioventricular coupling index’.

### 2.2. Inclusion and Exclusion Criteria

The following inclusion criteria were used: (1) studies related to the topic of the current review, (2) journal articles, (3) publications written in the English language, (4) studies including an adult population and (5) studies including a human population. We excluded studies that met any of the following exclusion criteria: (1) studies including children or animals, (2) conference proceedings, (3) supplementary materials and (4) abstracts without the full text available.

### 2.3. Literature Selection

**Figure 1:**
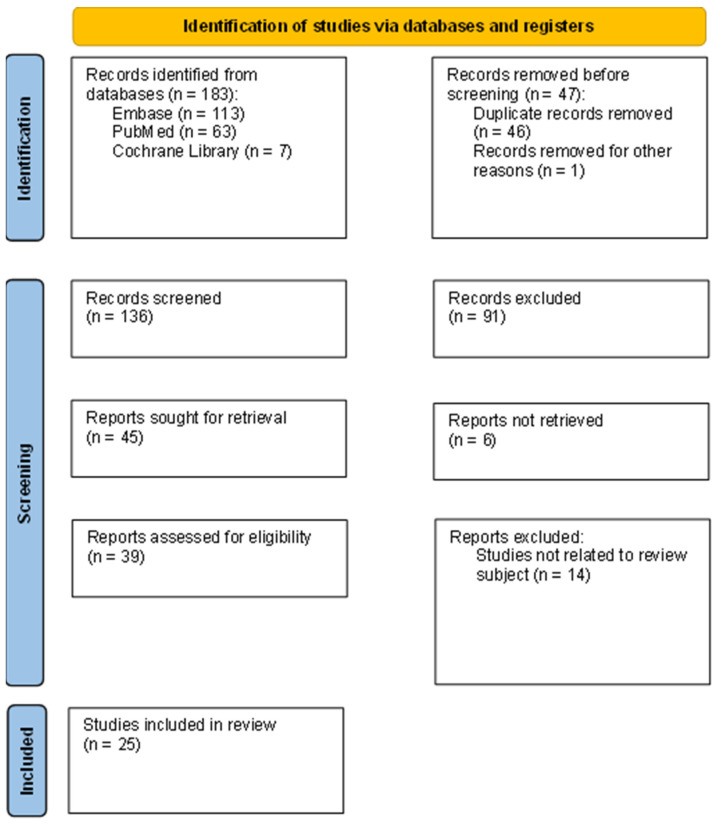
PRISMA flow diagram presenting process of study selection.

We assessed the most relevant studies about the LACI published up to the 15th of January 2025. Four researchers independently evaluated 183 articles according to the title, abstract, text and scientific validity. After removing duplicates (n = 46) and records that were not scientific texts (n = 1), 136 studies were initially screened, and 91 studies were removed due to meeting exclusion criteria or not meeting the inclusion criteria: abstracts without a full text available (n = 37), studies not related to the review subject (n = 36), studies in a language other than English (n = 0), studies including children (n = 3), studies including animals (n = 4) and duplicates (n = 11), while the other 45 studies were found to be appropriate for a full-text read and further assessment. Among these, 6 articles were not retrieved, and four reviewers read 39 full-text articles. In the end, 25 articles fulfilled the inclusion criteria. The entire selection process is presented in the PRISMA flow diagram in Figure 1.

## 3. Results and Discussion

### 3.1. Heart Imaging Techniques

The most commonly used cardiac imaging techniques are echocardiography, cardiac computed tomography and cardiac magnetic resonance. With the use of these techniques, multiple parameters useful in CVD diagnostics can be assessed. The advantages of these methods are their safety and non-invasiveness or minimal invasiveness, as well as the wide spectrum of diseases that can be diagnosed using them.

Echocardiography is a method of imaging based on ultrasound. It is widely used to assess the anatomical structure of the heart and its physiological activity. It allows for the measurement of parameters such as the left and right ventricles’ end-diastolic diameter (VEDD), the ventricles’ end-systolic diameter (VESD), the left ventricle mass (LVM), anatomical disorders in the valves and the presence of pathological masses. The physiological function is determined using parameters such as ejection fraction (EF), heart movement and filling pressure parameters, and valve parameters such as the diameter, pressure before and behind the valve and regurgitation jet. Examination is safe and does not use radiation, and TTE has no strong contraindications. TEE should not be performed in patients with esophageal varices or diverticulum of the esophagus if it is not necessary and if other methods could be performed. Echocardiography is an easy, relatively cheap and real-time examination that can be performed at the bedside; therefore, it is among the most valuable methods of cardiac imaging, especially in diagnosing valvular heart disease [4].

Cardiac computed tomography is a 3D method of imaging based on X-ray radiation and iodinated contrast injection. It is mostly used to determine the structure of the coronary arteries and valves and to calculate calcium score [5]. CT is not as widely available an imaging method as echocardiography, and requires appropriate preparation of the patient, which involves fasting for at least 8 h before examination and avoiding caffeine. It is contraindicated to perform CT in pregnant women, patients allergic to contrast and those with impaired kidney function. There is a small risk of adverse effects—allergic reactions to contrast, including anaphylactic shock, and complications associated with ionizing radiation, resulting in a low, but still existing, risk of development of neoplastic diseases, which is positively correlated with radiation dose and CT sites [6]. Despite these adverse effects, cardiac CT is considered the best non-invasive diagnostic method of coronary artery diseases and aortic disease [7]. Cardiac CT is one of the most valuable diagnostic methods in coronary artery disease (CAD) [8].

Magnetic resonance imaging (MRI) allows for the imaging of human tissues using a magnetic field, without exposure to ionizing radiation. Its disadvantages are low availability, a long duration of examination in comparison to other techniques and high cost. The parameters that can be assessed through CMR include the volumes of both the left ventricle (LV) and the right ventricle (RV) of the heart, ejection fractions, the LV mass, the presence of myocardial scars and abnormalities in regular wall motion [9]. With techniques like late gadolinium enhancement (LGE) and vasodilator stress CMR, even more precise examination can be performed. These techniques allow physicians to detect myocardial ischemia, predict myocardial viability and assess microvascular function. Nowadays, the increasing role of this method is observed in everyday clinical practice in cardiovascular diseases, based on still growing evidence for its utility in diagnostics, risk stratification and prognostics for several diseases of the heart, including heart failure (HF), ischemic heart disease, cardiomyopathies and valvular heart diseases. Table 1 compares the most important aspects of the mentioned imaging techniques.

### 3.2. Left Atrioventricular Coupling Index

**Figure 2:**
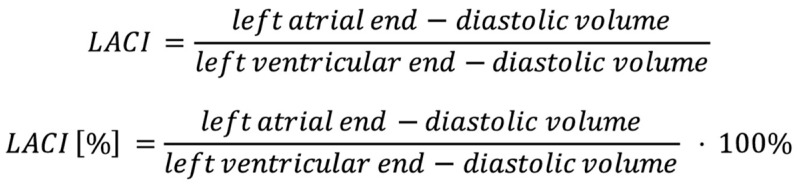
Formulas used for LACI calculation.

The left atrioventricular coupling index (LACI) is a novel parameter that is a potentially useful prognostic marker in several cardiac diseases, including heart failure (HF), atrial fibrillation (AF) and coronary artery disease (CAD) [10]. The LACI may be useful in the prediction of cardiovascular events and death due to cardiovascular conditions. It appears that the LACI, as a parameter that reflects left atrioventricular coupling, has better predictive value than atrial and ventricular markers used individually, as conventional parameters, including the LVEF, LVMI, LAVI and LV GLS, assess only the function of one chamber of the heart, and do not take into account cooperation with other chambers. The LACI overcomes this limitation by simultaneously assessing the function of both the LA and LV and their interactions. The LACI is defined as the ratio of left atrial end-diastolic volume to left ventricular end-diastolic volume, and is usually presented in percentages. Higher values of the LACI are a marker of left atrioventricular uncoupling. The equation used to calculate the LACI is presented in Figure 2.

The volumes of both the left atrium (LA) and the left ventricle (LV) are measured in the same end-diastolic phase of the cardiac cycle. Left atrial end-diastolic volume is calculated with the use of the Biplane formula, and the same goes for each of the imaging methods. The formula requires measurements from two-chamber and four-chamber imaging, while the left ventricular end-diastolic volume is measured from the stack of short-axis cine images. Echocardiography, cardiac CT and CMR can all be used in the measurement of LACI components [10,11,12]. The left atrial end-diastolic volume is calculated after mitral valve closure, or, with use of an electrocardiogram, during the QRS peak. In some protocols, the LACI can be calculated semi-automatically, where the operator outlines the border of the heart chamber and an automatic system tracks its movement during the cardiac cycle [13]. In other protocols, mostly used in echocardiography, the system automatically delineates the borders of the heart chambers, while the operator has a chance to correct them before automatic tracking [12]. Moreover, the LACI can be calculated fully automatically with the usage of artificial intelligence algorithms during CMR examination, with a dice score of 89.4 for LV end-diastolic volume and 87.9 for LA end-diastolic volume [14]. Additionally, there is an equivalent of the LACI for the right chambers of the heart, called the right atrioventricular coupling index (RACI), and the combined atrioventricular coupling index (CACI) is the sum of the LACI and RACI [15]. Currently, there are no studies comparing the calculation of the LACI by different methods of imaging, although this seems to be essential in order to procure a better understanding of the LACI, to determine its cut-off values and to enable wider use of the LACI [16]. According to Tim Leiner, the LACI might be a promising parameter to calculate during routine cardiological appointment if it could be calculated with the use of TTE [17]. Echocardiography is still cheaper and a significantly more common method of heart imaging than CMR and cardiac CT. Additionally, the lack of adverse effects from contrast or radiation and the possibility of bedside examination promote echocardiography as the best method of calculating the LACI, popularizing this parameter as a predictor of cardiological diseases.

Some studies have examined the prognostic value of the LACI. Pezel et al. [10] investigated the LACI in people with no clinically recognized cardiovascular diseases. The data collected in the Multi-Ethnic Study of Atherosclerosis (MESA) were used. The participants, aged 45 to 84 years, were examined with CMR. The mean follow-up period was 13.0 years. In this study, a positive association was found between the LACI and the incidence of AF, HF and coronary heart disease death. Moreover, a significant association was found between the LACI and hard cardiovascular diseases, which were defined as myocardial infarction, stroke, resuscitated cardiac arrest and death caused by coronary disease. The incidence of all previously mentioned conditions was higher in the fourth quartile (>22.4%) than in the first quartile (<11.4%) of LACI values.

Another study aimed to find an association between the LACI and cardiovascular death [11]. The study included 1444 participants without cardiovascular diseases, who underwent cardiac CT examination. The median follow-up period was 6.8 years. The annual occurrence of cardiovascular death gradually increased with increasing values of the LACI in subsequent terciles. It was found that the LACI was independently associated with cardiovascular and all-cause death in the studied population. Participants with an LACI ≥ 25% had a greater risk of cardiovascular death than those with an LACI lower than 25%, based on multivariable analysis. Moreover, it was found that implementation of the LACI may improve the prognostic value of examination when used in addition to other risk factors. The importance of LACI assessment in specific disease units will be presented in the following sections of the current review. The study’s limitations were a lack of information about medications used by patients and a lack of calcium score assessment in all participants.

### 3.3. The Role of the Left Atrioventricular Coupling Index in Heart Failure

Heart failure (HF) is a clinical syndrome caused by reduced cardiac output or elevated intracardiac pressure. Due to HF, the heart is unable to pump enough blood to meet the organism’s need for oxygen. The incidence of HF is constantly rising in developed countries, due to aging populations. HF greatly reduces the quality of life of affected patients, and is associated with significant expenses for national healthcare systems [18,19]. Moreover, HF is characterized by a very high 5-year mortality of about 50%, which makes its prognosis worse than in most malignant neoplasms. Numerous studies have investigated the importance of LACI assessment in HF patients.

In Backhaus et al.’s research [20], 68 patients with exertional dyspnea and symptoms of diastolic dysfunction were examined. Based on pulmonary capillary wedge pressure (PCWP), patients were divided into HFpEF (n = 34) and noncardiac dyspnea (n = 34) groups. All patients underwent CMR examination, both at rest and during an exercise stress test. The LACI was significantly higher in the HFpEF group than in the noncardiac dyspnoea group. Moreover, a correlation was found between the LACI and PCPW, both at rest and during the exercise stress test. The LACI carried the highest diagnostic accuracy in identifying invasively verified HFpEF. The LACI was able to discriminate between HFpEF and noncardiac dyspnea. The performance of the LACI was similar between rest and exercise stress tests, in contrast to conventional functional parameters, which gained greater accuracy during stress testing. Moreover, examination revealed that elevated LACI values were associated with an increased risk of hospitalization within 24 months, which supports the LACI’s usefulness as a prognostic marker. However, Backhaus et al.’s research has some limitations. The study involved only a small group of participants, and it was a single-center study. The study would benefit from a larger number of included patients.

Another study, conducted by Pezel et al. [21], aimed to determine the prognostic value of the LACI and annual changes in the LACI (ΔLACI) in the prediction of HF. The authors used data collected in the MESA study. The research included 2250 patients, aged between 45 and 84 years, with no clinically recognized CVD. Participants were examined by CMR at the baseline, and underwent a second CMR examination after a mean time of 9.6 years. The mean follow-up was 6.8 years after the second CMR examination. The study concluded that an LACI above the cut-off value of 30% and a ΔLACI above 1.5%/year were associated with HF incidents. The study of the above-mentioned indices revealed a higher prognostic value than traditional risk factors.

The LACI may also have diagnostic and prognostic utility in different HF phenotypes. Lange et al.’s study [22] included 73 participants, of which 22 had heart failure with preserved ejection fraction (HFpEF), 17 had heart failure with mildly reduced ejection fraction (HFmrEF), 15 had heart failure with reduced ejection fraction (HFrEF) and 19 people were healthy volunteers. In the group of patients with HFpEF, the LACI was significantly increased when compared to the healthy group, while there were no differences in the LACI between the healthy and HFmrEF and HFrEF groups. There were no significant differences in LACI values between HFmrEF and HFrEF. Moreover, patients with HFpEF presented disproportional enlargement of the LA when compared to the volume of the LV. The study revealed that the LACI may be used to differentiate patients with different phenotypes of HF. However, the study has some limitations. Firstly, the studied group was of a small size, and there is a risk of the results being improperly generalized. Due to the exclusion criteria, selection bias might exist. Moreover, the patients who took part in the study were not always age-matched, and HF etiology, comorbidities and individual traits of patients were not taken into consideration by the authors.

Other researchers have tried to assess the relevance of the LACI calculated based on echocardiographic measurements in HF [12]. The study included 60 HF patients divided into HFpEF (LVEF ≥ 50%) and HFrEF (defined in the study as LVEF < 50%) subgroups, and a control group consisting of 100 healthy people. The measurement of LACI components was performed with echocardiography in two-chamber and four-chamber views, and the volumes of the LA and LV were calculated using the biplane Simpson’s method. Differences in the LACI were observed between the HFpEF, HFrEF and control groups. The HF group had higher values of the LACI than the control group, while the HFpEF group had significantly higher LACI values than the HFrEF group. Moreover, the LACI had a significant correlation with other echocardiographic parameters: the LVEF, LAVI, LVMI and LV GLS. In multivariate analysis, the LACI was also found to be an independent predictor of HFpEF, and the authors claimed that the LACI is potentially useful in HFpEF diagnostics. The main limitations of this study are the small group of included patients and the fact that it was a single-center study.

Furthermore, in another study [14], the authors tried to assess the usefulness of the LACI calculated by artificial intelligence algorithms during CMR examination. The study included 2134 patients, half of whom had normal vasodilator stress CMR, while the other half had abnormal vasodilator stress CMR, defined as the presence of inducible ischemia or LGE. The population was 1:1 propensity score-matched. The authors examined the association between the LACI and primary outcomes, including hospitalization due to acute heart failure (AHF) and cardiovascular death, as well as secondary outcomes, including cardiovascular and all-cause death, non-fatal myocardial infarction, ventricular tachyarrhythmias, incident HF and late coronary revascularization. The patients in both groups underwent vasodilator stress CMR, and the median follow-up was 5.2 years. The LACI was calculated using artificial intelligence algorithms. It was found that patients with high values of the LACI had more primary outcomes annually than those with low values of the LACI when the patients were divided into terciles. An association between the LACI and primary outcomes was found in patients with normal and abnormal stress CMR and in the overall population. An LACI ≥ 25% was independently associated with the occurrence of primary outcomes in these populations. The inclusion of stress CMR and the LACI also significantly improved the C-index in the prediction of primary outcomes from 0.60 to 0.76. Among the secondary outcomes, the LACI was associated with the number of AHF hospitalizations and cardiovascular and all-cause deaths. All of these findings suggest that the LACI is a promising parameter that can be used in the prediction of outcomes in patients with HF, including patients with both normal and abnormal CMR. The weaknesses of this study include that it was a retrospective, single-center study. Moreover, data on the medications used by patients were not collected, and the volume of the LA could have been underestimated, due to the used measurement method.

It appears that the LACI has several uses in patients suffering from HF. The LACI may be a useful parameter in the diagnosis of HF, especially in HFpEF. Moreover, the LACI and its derivative, the ΔLACI, may be used in the prediction of HF. Furthermore, the LACI may be potentially useful as a prognostic marker and a marker of possible future exacerbation of HF. It appears that echocardiography can be used instead of CMR in HF patients to assess the LACI. The possibility of relying on echocardiography instead of CMR in determining the LACI would make these measurements more accessible, which is important, due to the constantly growing number of patients suffering from HF. However, there are not much data currently available on this subject, so further studies are needed to support this statement. These studies should include a larger number of patients.

### 3.4. The Role of Left Atrioventricular Coupling Index in Atrial Fibrillation

Atrial fibrillation (AF) is the most common supraventricular sustained tachyarrhythmia, with the rate increasing with age. AF disrupts the proper functioning of the atria and causes ineffective blood flow to the heart ventricles [23,24,25]. Another effect of AF is irregular contractions of the heart’s ventricles. The symptoms of AF include heart palpitations, irregular heartbeat, fatigue, shortness of breath and chest pain. AF can also cause dangerous thromboembolic complications, including stroke. AF can usually be diagnosed based on an ECG and Holter monitoring. It has also been found that the LACI and annual changes in the LACI (ΔLACI) are independent predictors of future AF incidents in people who have not previously suffered from a cardiovascular disease [26].

Pezel et al. [26] investigated the usefulness of the LACI and ΔLACI using data collected prospectively during the MESA study. The study included 1911 participants aged 45 to 84 who underwent CMR examinations and had no clinically recognized cardiovascular diseases. The second CMR examination took place 10 years after the first one, and the mean follow-up was 4 years after the second CMR examination. An LACI ≥ 30% was associated with AF occurrence. Patients who experienced AF also had a higher LV mass and volume, and lower left atrial functional parameters, than those without AF incidents. Moreover, an ΔLACI ≥ 1.5% was also associated with AF in this population. After adjustment, both the LACI and ΔLACI were independently positively correlated with AF occurrence. Based on a multivariable model, these parameters also had a greater predictive value for AF than the Cohort for Heart and Aging Research in Genomic Epidemiology–Atrial Fibrillation (CHARGE-AF) score and individual left atrial and left ventricular parameters. Another fact worth noting is that participants who developed AF had a greater increase in the volume of LA than participants without AF. Meanwhile, the decrease in LV volume was similar in both groups. The drawbacks of this study include the following: the LA volume was measured using 2D methods, and its volume could have been underestimated; only asymptomatic participants were included in the study; furthermore, assignment to the AF group was based on discharge codes, so some of the AF incidents could have been overlooked; and the ΔLACI was an average value, based on two CMR examinations conducted with an interval of 10 years. Moreover, the authors also claimed that the LACI may not be precise in patients with an enlarged LA and LV due to structural heart diseases.

Another study [27] investigated the usefulness of the LACI in the prediction of paroxysmal atrial fibrillation (PAF) recurrence. Patients with PAF are often treated with catheter ablation (CA); however, the possibility of recurrence following surgery is high. The cited study included 164 patients with PAF; during the 12-month follow-up after ablation, 56 of them had experienced AF recurrence. Using a multivariable logistic regression model, it was found that a high LACI value was significantly associated with an increased risk of AF recurrence, and that the LACI can be used to identify patients with increased risk of AF recurrence. The cut-off value was set to 34.5%, using calculation of the Youden Index, providing a sensitivity of 75.9% and specificity of 69.6%. During this study, it was found that the LACI is a predictive factor for the recurrence of PAF, and is a better predictor than traditional parameters, such as LA and LV volume. There were numerous limitations to the cited study. Firstly, the study was performed in a single center, and included a relatively small group of patients. The follow-up period of this study could also have had an impact on the results, as it lasted only 12 months. The cited study would benefit from a longer follow-up period. Another limitation is associated with Holter examination being performed only two times per month, and so probably not every recurrence of PAF was observed.

The LACI and ΔLACI seem to be promising parameters in the prediction of AF occurrence; however, more studies are required to confirm their usefulness. Moreover, standards for these indicators should be established, including standards for different age groups. As suggested in Teis and Delgado’s editorial, future studies correlating the LACI with invasive measurement could be useful in determining the cut-off value of the LACI [9]. Furthermore, a similar study including participants with cardiovascular diseases should be performed to assess the usefulness of the LACI in this group, as suggested by Leiner [17]. Also, future studies should include other imaging techniques, apart from CMR, to assess whether these methods could also be used in the measurement of LACI components used for AF prediction, as CMR examination is less available, more expensive and more time-consuming than other heart imaging methods. It also seems interesting to investigate whether the expected result of pharmacotherapy or cardioversion in the treatment of AF and the risk of AF recurrence can be assessed by calculating the LACI. Perhaps it would also be valuable to investigate whether the LACI could be used in the assessment of the risk of complications of AF, such as stroke.

### 3.5. The Role of Left Atrioventricular Coupling Index in Diabetes Mellitus and Hypertension

Diabetes mellitus (DM) and hypertension (HTN) are some of the most prevalent diseases in the world. There are around 830 million patients suffering from diabetes and 1.28 billion patients with hypertension. According to Naseri et al.’s study [28], 70% of patients with diabetes have increased blood pressure, which illustrates the relationship between both diseases. DM and HTN are one of the major risk factors for CVD. The most frequent cardiovascular complications of those diseases include coronary artery disease (CAD) and HF [29,30]. DM often alters the structure and function of cardiac muscle. In patients with DM, cardiovascular complications are a leading cause of mortality and disability—about 70% of patients with DM die because of CVD [31], which acknowledges the importance of prevention and proper cardiovascular diagnostics in DM.

In one retrospective study [32], the LACI was determined by CMR in a population consisting of 35 prediabetic patients, 32 diabetic patients and 84 healthy people. The differentiating factor for the study populations was the level of HbA1C. The study revealed no significant correlation between the LACI and HbA1C in a univariate model of analysis. Furthermore, there was no significant correlation between left atrial deformation parameters and the LACI. Researchers disqualified patients with diagnosed CVD because including them in the study would disturb the results. However, when analyzing this study, it is important to consider its weak points, the main one being the small sample size.

Another study [33] involved 111 patients, divided into two groups: 59 healthy persons and 52 patients with DM. The study was conducted to evaluate cardiac stiffness. The LACI values, calculated based on echocardiographic examination, in patients with DM type 2 were significantly higher compared to in the control group—17.12%  ±  6.72% and 12.28%  ±  3.96%, respectively. During the study, correlations were found between the LACI and left atrial and left ventricular stiffness. These parameters were also elevated in the diabetes group. The study highlights that the LACI, combined with LA and LV stiffness parameters, has prognostic value for detecting cardiac changes in patients with DM type 2. These markers can become useful prognostic tools for cardiovascular risk. However, the cited study has several limitations. Firstly, it was a single-center observational study with a relatively small group of participants. Moreover, the studied group included only Vietnamese people, so selection bias might have been present, and generalization of the study results is not possible. Furthermore, the LACI was assessed only once. The change in the LACI over time was not observed, as the study had no follow-up. In addition, the patients were still using medications before the examination, which could have had a potential impact on the results.

Patients with DM and hypertension have higher LACI values compared to patients with DM and no hypertension [34], which was revealed in a retrospective study with the use of CMR feature tracking. The studied population consisted of 103 patients with diabetes and hypertension and 73 patients with diabetes only. In the group with both disorders, the population had significantly higher BSA, a higher smoking rate and lower total cholesterol, but researchers claimed that they adjusted for these differences. The LACI in the group with hypertension was significantly higher than in the group with only diabetes, which was confirmed in multivariate analysis. The main limitations of the study are the small study group and its retrospective character. To confirm that hypertension is an independent factor influencing the LACI in a population of diabetes patients, a consecutive multi-center prospective study with a larger study group is essential.

Furthermore, the LACI was decreased in patients with resistant hypertension (RHTN) after using spironolactone [35]. In Girard et al.’s prospective study, 47 patients with RHTN were subjected to spironolactone usage to ascertain its influence on heart structure. CMR examination was performed initially and after 6 months of study. The study revealed that the LACI significantly decreased from 28.2% ± 11.5% to 22.7% ± 9.2%. The LACI was significantly correlated with the LVMI, which was reduced during spironolactone intake. Researchers found that spironolactone’s important role in improving atrioventricular coupling might be associated with its diuretic effect and decreasing levels of BNP. The lack of a control group, small population and short observation time are the main issues of the study. The LACI decrease might also have been an independent effect of lowering blood pressure, but the authors did not exclude this. In this case, research on spironolactone’s effect on cardiac muscle activity should be continued.

In conclusion, studies assessing the role of the LACI in DM have conflicting results. Moreover, these studies rely on small study groups, so it is impossible to assess whether LACI calculation is useful in this disease. The data on LACI assessment in HTN are also limited. More research is needed to determine whether assessing the LACI in DM and HTN provides any valuable information. Prospective studies with a long follow-up period and many participants would be most valuable.

### 3.6. The Role of the Left Atrioventricular Coupling Index in Other Conditions

Currently available research also indicates the potential use of the LACI in conditions other than HF, AF, DM and hypertension. These conditions include cardiomyopathies, myocarditis, myocardial infarction, beta-thalassemia major, metabolic syndrome and light-chain amyloidosis.

#### 3.6.1. Cardiomyopathies and Myocarditis

Patients with hypertrophic cardiomyopathy (HCM) have a considerable chance of developing AF, which significantly raises their mortality. Since this disease affects mostly the wall of the heart’s left ventricle, investigation of the LACI in this disease was performed. A study on a population of 373 patients with HCM and without a history of AF was conducted [36]. The LACI was calculated based on measurements performed using transthoracic echocardiography. The median follow-up period was 11 years. During follow-up, 118 cases of new-onset AF were documented. The research revealed, using multivariable regression models, that higher values of the LACI are associated with increased risk of new-onset AF, and the LACI showed better predictive value than commonly used LA parameters. Furthermore, patients with an LACI below the cut-off value of 40% had a higher cumulative time before experiencing cardiovascular events. However, this study had some limitations, including possible selection bias and its retrospective character.

Another disease affecting mostly the left ventricle, for which the LACI might have prognostic value, is dilated cardiomyopathy (DCM). Although DCM mostly affects the LV, parameters such as the LAVI and late diastolic mitral annular velocity (A’), which reflect the function of the LA, can be used in the assessment of LV diastolic dysfunction [37]. Furthermore, focused analyses have proved atrial indexes such as the LAVI and the annual change in LAVI (ΔLAVI) to be relevant risk factors of death, heart transplantation and heart failure in patients with DCM. This association suggests that longitudinal assessment of left atrial dimensions should be further explored, as its predictive value might be proven an important prognostic factor in heart diseases [38]. Since the LACI takes into consideration both LV and LA function, it could be a useful predictor of MACE in DCM. Vîjîiac et al.’s study [15] included 121 patients with DCM. The study aimed to determine whether the LACI and other atrioventricular coupling indices, like the right atrioventricular coupling index (RACI) and the combined atrioventricular coupling index (CACI), have any predictive value in the prognosis of major adverse clinical events (MACEs) in patients with DCM. The RACI was defined as the ratio between the right atrial end-diastolic volume and right ventricular end-diastolic volume, and the CACI as the sum of the RACI and LACI. Measurements were taken using echocardiography. A total of 55 participants reached the endpoint of the research, which was death, non-fatal cardiac arrest, heart transplant and readmission to the hospital due to HF exacerbation. The study concluded that all three indices were significantly elevated in patients with MACEs, and all proved to be independent predictors of MACEs in univariable analysis and a multivariable model of analysis. The cut-off values for the studied indices were as follows: 20% for the LACI, 21% for the RACI and 44% for the CACI. The cited study revealed that, although the LACI can be an independent predictor of MACEs, the CACI had better predictive value, and should be further analyzed in future studies. The primary limitations of this study were a relatively small population and a relatively short follow-up period. Furthermore, it was a single-center study and patients with AF were excluded, leading to potential selection bias. Another limitation mentioned by the authors is that, due to lack of reference values of the LACI, RACI and CACI in the general population, the determined cut-off values apply only to DCM, and not to any other diseases.

Patients who suffer from myocarditis may not fully recover, and the disease can progress to DCM, increasing their mortality due to increased risk of heart failure and life-threatening arrhythmia. Since the prognosis of myocarditis is based on MRI examination, and one of the predictors is LV strain, the LACI might also prove to be an important predicting factor. Chen et al.’s. study [39] investigated the prognostic value of the LACI in patients with suspected myocarditis and preserved LVEF. The research included 165 participants with suspected myocarditis who underwent MR examination. The median follow-up time was 917 days, and during this time, 23 of the patients experienced MACEs. During univariable and multivariable Cox analyses, the LACI, although it reflects the correlation between the LV and LA, did not prove to be associated with the occurrence of MACEs, which stands in contrast to previous studies. The cited study has some important limitations. The study had a retrospective character, three different MR scanners were used in the study and the number of patients included in the research was relatively small.

#### 3.6.2. Myocardial Infarction

Lange et al. investigated the potential of the LACI to provide prognostic value to predict MACEs, including death, reinfarction and HF development, within 12 months after an AMI [13]. The study involved 1046 patients after AMI, among whom 719 had ST-segment elevation myocardial infarction (STEMI) and 325 had non-ST-segment elevation myocardial infarction (NSTEMI). The patients were treated with primary percutaneous coronary intervention (PCI). Participants underwent CMR imaging within 10 days after AMI. During the 1-year-long follow-up, 73 MACEs were documented, including 34 deaths, 18 reinfarctions and 21 patients who developed HF. It was observed that patients with MACEs had significantly higher LACI values than those without MACEs. The LACI cut-off value was set to 34.7%, using the Youden Index, to differentiate between high- and low-risk patients. Univariable regression analyses and multivariable Cox regression, including baseline confounders and the LVEF, revealed significant associations between the LACI and MACEs. After inclusion of the LV GLS and LA total strain in the multivariable Cox model, the association between the LACI and MACEs was not present anymore. Among the endpoints, the LACI was significantly associated with death and HF, while its association with reinfarction was not significant. This study revealed that the LACI is a sensitive and specific prognostic marker in patients following AMI. However, the study included some shortcomings. The selection bias might be present in the study as only stable patients were included in the study. The selection of the patients probably led to a decreased number of MACEs when compared to the entire population of patients after AMI. Furthermore, different MR scanners were used in the study, although every center followed the same study protocol. The study also did not define the optimal time for performing CMR after MI.

#### 3.6.3. Beta-Thalassemia Major

In patients with beta-thalassemia major, the LACI seems to be significantly higher, which was stated in Meloni et al.’s cross-sectional study [40]. A group of 292 patients with beta-thalassemia major was compared with a group of 32 patients without beta-thalassemia. The LACI was calculated using parameters of heart function measured using CMR. In the healthy population, the LACI was significantly lower when compared to the population with beta-thalassemia. Furthermore, beta-thalassemia patients after splenectomy presented significantly elevated LACI values compared to patients with a spleen. Additionally, 14.5% of beta-thalassemia patients with diagnosed DM revealed higher LACI values than patients without this disorder (21.24 ± 10.85%). As the study revealed, a higher LACI was associated with older age, a higher LGE and more frequent occurrence of cardiac complications, such as heart failure, arrhythmia and pulmonary hypertension. It was determined that an LACI >23.6% correlated with a more common appearance of cardiac complications, with a specificity of 75.8% and a sensitivity of 74.1%. Associations with significant correlation in a univariate model were confirmed in a multivariate model. The LACI seems to be useful in the prognosis of cardiac complications in patients with beta-thalassemia major, but successive studies with larger study groups are needed.

#### 3.6.4. Metabolic Syndrome

Two studies on the influence of metabolic syndrome (MetS) on the LACI were recently released. In Huang et al.’s observational study [41], it was stated that patients with MetS had a higher LACI than the healthy population. A total of 179 patients with metabolic syndrome were divided into two subgroups, with or without coexisting metabolic dysfunction-associated fatty liver disease (MAFLD), and were compared with 81 controls without metabolic syndrome. The LACI was calculated using measurements taken using CMR. The results presented significantly higher LACI values in the study group, and higher LACI values in the subgroup with MAFLD (17.2%), compared to the subgroup without MAFLD (15.8%), based on multivariable analysis. The study had a small study group and retrospective character, which, without further analysis, do not allow for the establishment of the prognostic role of the LACI in MAFLD. On the contrary, another study did not confirm this correlation [42]. A group of 181 patients after myocardial infarction (MI) and a group of 107 controls were divided into subgroups with metabolic syndrome (MetS) and without MetS. CMR was performed to evaluate the LACI and other parameters of cardiac work. Patients after MI had significantly higher LACI values than the control group, but there was no significant correlation between the LACI and the presence of metabolic syndrome in the MI group, as well as in the control group, confirmed in a multivariable model of analysis. As in the study above, the study group was small and the study lacked follow-up. Additionally, Liu et al. diagnosed metabolic syndrome not by the main definition of the International Diabetes Federation, but by replacing the waist circumference criterion with BMI. This is acceptable in modified IDF criteria, but to evaluate the significance of the LACI in MetS and avoid false results, a diagnosis of metabolic syndrome should be performed according to the main criteria.

#### 3.6.5. Light-Chain Amyloidosis

The LACI may also have a potential use as a prognostic marker in light-chain amyloidosis. In a study conducted by Wang et al. [43], 179 participants suffering from light-chain amyloidosis underwent CMR examination to assess the impact of the LACI on all-cause mortality. The mean follow-up period was 30 months. Patients with cardiac amyloidosis had a higher LACI than patients without cardiac amyloidosis. The LACI showed higher values with increasing Mayo stages and NYHA classes; however, this association did not reach statistical significance. The LACI was significantly higher in patients who died in comparison to patients who survived the follow-up period. Basing on multivariate Cox regression, an LACI ≥ 49.3% was an independent all-cause mortality predictor in light-chain amyloidosis in patients with Mayo stages IIIa and IIIb; however, this cut-off value had no significant impact on outcomes in patients with Mayo stages I and II. The authors claimed that the LACI can be used in risk assessment in advanced light-chain amyloidosis patients, in addition to the Mayo stage assessment. The study has some limitations including, that it was a single-center study and had a small number of participants. The LA volume was measured using the 2D method, possibly underestimating the volume. Moreover, the study focused only on patients’ deaths, and it did not provide data on other cardiac events.

#### 3.6.6. Sodium Intake

As a measure of sodium intake, 24 h urinary sodium correlates with the LACI [44]. In a cross-sectional study of 398 patients divided into three groups depending on urinary sodium level (low, moderate, high), the LACI was significantly greater (25.92% ± 15.45%) in the high-urinary-sodium than in the low-urinary-sodium group (18.73% ± 10.10%), which was stated in multivariable analysis. The group of patients with high urinary sodium had significantly greater blood pressure (both diastolic and systolic), a higher BMI, a higher percentage of smokers and alcohol users and more frequent obesity. The researchers noted the unreliability of 24 h urinary sodium as an indicator of sodium intake, thus, a new marker or method of research is needed. Prospective multi-center studies are necessary to determine the correlation between the LACI and sodium intake.

#### 3.6.7. Menopause

The LACI correlated with the occurrence of heart failure, atrial fibrillation, coronary heart disease deaths and severe CVD in a perimenopausal population [45]. In a prospective MESA study, 2087 women were divided into premenopausal and postmenopausal groups. Menopause status was declared by participants, and confirmed by researchers with the use of an algorithm based on age and previously undergone procedures inducing menopause. The postmenopausal group was divided into a subgroup with hormone therapy and a subgroup without hormone therapy. The LACI was calculated based on CMR examination, and the study group was observed for over 13 years. The study showed that the postmenopausal population had significantly higher LACI values than the premenopausal women. Additionally, the subgroup with hormone therapy had significantly lower LACI values than the subgroup not using hormone therapy. The significance of these results was confirmed in multivariable analysis. The LACI was positively correlated with biochemical changes occurring during menopause, increasing total testosterone and DHEA and lowering estradiol, but correlations with free testosterone and SHBG were not significant. The study demonstrated that an LACI higher than 25% can be an independent marker of AF, HF, CHD death and severe CVD. The primary limitation of the study was its healthy study group, which may have caused the results to not correlate with the general population. Nevertheless, the study had several strengths, such as its prospective character and the involvement of a large group of participants.

### 3.7. The Cut-Off Value of the Left Atrioventricular Coupling Index

Authors of studies on the LACI and its changes in particular diseases have determined cut-off values for the LACI in the prediction of cardiovascular complications, which were established by selecting the highest AUC on the ROC curve. Pezel et al. defined an LACI above 30% as a prognostic factor of heart failure [21], while Dang et al. suggested that for diagnosing HFpEF, the LACI should reach 33.07% [12]. A cut-off value set above 30% can predict the occurrence of AF [26]. Additionally, in another study, it was found that an LACI above 34.5% could be a predictor of recurrence of AF [27]. Meucci et al. set a cut-off value above 40% for patients with HCM to determine a group of patients with a shorter time to cardiovascular events [36], while in patients with DCM, an LACI above 20% was found to be a prognostic factor of higher risk of MACEs [15]. Meloni et al. determined 23.6% as the cut-off value for the LACI in patients with beta-thalassemia major, which could be used for predicting cardiac complications [40]. In conclusion, there is no specific cut-off value for the LACI which could be generally used to determine groups of patients with higher risk of cardiovascular disease. Each condition may modify the LACI at a different level; therefore, further studies are required to standardize the cut-off value of the LACI. Up until now, a lack of standardization and discrepancies in study results are the main limitations that preclude the common use of LACI. In summary, the LACI can be measured using different imaging techniques, including CMR, cardiac CT and echocardiography, which vary in imaging accuracy. Currently, there are no studies comparing LACI values calculated based on these methods, which creates another problem in establishing a standardized cut-off value for the LACI. The LACI should probably be defined as the MRI-LACI, CT-LACI, etc.

## 4. Conclusions

**Figure 3:**
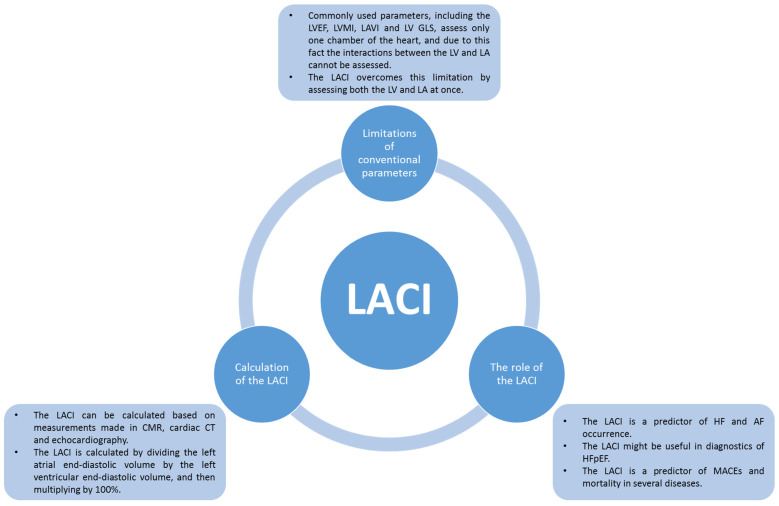
Most important information about LACI.

In conclusion, the LACI is a promising parameter that can be easily assessed in different imaging techniques. The LACI seems to have use in diagnostics and prognostics of several diseases, especially in HF and AF. It appears that higher values of the LACI are associated with greater risk to people in terms of cardiovascular events and the development of diseases like HF and AF. However, currently available data are limited in terms of other diseases, e.g., DM and HTN. Moreover, most studies include only small study groups, and most studies with larger groups rely on a single cohort (MESA). Furthermore, the cut-off values of the LACI vary between studies. Also, most existing studies use CMR to assess the LACI, and not many studies include other imaging techniques. The most important findings regarding the LACI are presented in Table 2 and Figure 3.

Further studies on the LACI are required. These studies should have a greater number of participants and rely on different cohorts. Moreover, more studies should include echocardiography, as this method is more available than CMR. Echocardiography can be widely used, and due to this accessibility, the findings regarding the LACI would benefit more patients. A study comparing LACI values based on CMR and LACI values based on echocardiography in the same population would be especially valuable.

## Figures and Tables

**Table 1:** Comparison between cardiac imaging techniques.

Feature	Echocardiography	Cardiac CT	Cardiac MR
Accessibility	Very high	High	Low
Bedside examination	Yes	No	No
Real-time examination	Yes	No	No
Time of examination	Short	Short	Long
Contrast	No	Yes, iodinated	Yes, gadolinium
Ionizing radiation	No	Yes	No
Safe in pregnancy	Yes	No	Yes
Cost	Cheap	Moderate	Expensive

**Table 2:** The main findings regarding the LACI in the studies included in the current review. The most important findings have been bolded.

Authors and Year of Publication	Imaging Technique	Studied Group	Main Findings	References
Pezel et al., 2021	CMR	People without recognized cardiovascular diseases (MESA population)	The LACI is a predictor of HF, AF, coronary heart disease death and hard cardiovascular disease in people without recognized cardiovascular diseases. The LACI has greater prognostic value than LA and LV parameters individually.	[10]
Pezel et al., 2023	Cardiac CT	People without recognized cardiovascular diseases (MESA population)	The LACI is independently associated with cardiovascular and all-cause death in people without cardiovascular diseases.	[11]
Backhaus et al., 2023	CMR	Patients with HFpEF or noncardiac dyspnoea	The LACI can distinguish patients with HFpEF from patients with noncardiac dyspnoea. A correlation was found between the LACI and PCPW during rest and exercise. High values of the LACI are associated with an increased risk of hospitalization within 24 months.	[20]
Pezel et al., 2021	CMR	People without recognized cardiovascular diseases (MESA population)	Elevated LACI and ΔLACI values are associated with HF incidents.	[21]
Lange et al., 2024	CMR	Patients with heart failure	The LACI was increased in patients with HFpEF when compared to healthy controls. No difference in the LACI was found between HFmrEF and HFrEF patients and healthy controls.	[22]
Dang et al., 2024	Echocardiography	Patients with heart failure	Patients with HF have increased LACI variability when compared to healthy people. LACI variability is highest in patients with HFpEF. The LACI may have use in HFpEF diagnostics.	[12]
Pezel et al., 2023	CMR	Patients with normal or abnormal stress CMR	The LACI is independently associated with cardiovascular death and hospitalization due to acute HF in patients with normal or abnormal stress CMR.	[14]
Pezel et al., 2022	CMR	People without recognized cardiovascular diseases (MESA population)	The LACI and ΔLACI were predictors for AF occurrence in a population without diagnosed CVD. The LACI and ΔLACI have greater predictive value than traditional parameters in predicting AF.	[26]
Li et al., 2024	Echocardiography	Patients with paroxysmal atrial fibrillation after catheter ablation	A high value of the LACI is significantly associated with a higher risk of AF recurrence after catheter ablation. The LACI is a better predictor of AF recurrence after catheter ablation than traditional parameters.	[27]
Zhou et al., 2024	CMR	Patients with prediabetes and diabetes	The LACI does not significantly correlate with HbA1C level. The LACI does not significantly correlate with LA deformation parameters.	[32]
Dang et al., 2024	Echocardiography	Patients with diabetes	LACI values were significantly higher in patients with DM type 2 when compared to healthy people. The LACI correlates with left atrial and left ventricular stiffness. The LACI has prognostic value for detecting cardiac changes in patients with DM type 2.	[33]
Shi et al., 2023	CMR	Patients with diabetes with and without coexisting hypertension	The LACI was significantly higher in patients with coexisting hypertension in a population of diabetic patients.	[34]
Girard et al., 2023	CMR	Patients with resistant hypertension using spironolactone	The LACI was significantly lower after using spironolactone in patients with resistant hypertension. There is a positive significant correlation between the LACI and LV mass.	[35]
Meucci et al., 2022	Echocardiography	Patients with hypertrophic cardiomyopathy	The LACI is associated with an increased risk of AF in patients with HCM.	[36]
Vîjîiac et al., 2024	Echocardiography	Patients with dilated cardiomyopathy	The LACI was elevated in patients with DCM who experienced MACEs, and can be an independent predictor of MACEs.	[15]
Chen et al., 2024	CMR	Patients with suspected myocarditis and preserved LVEF	The LACI is not associated with MACEs in patients with suspected myocarditis.	[39]
Lange et al., 2023	CMR	Patients after acute myocardial infarction treated with primary percutaneous coronary intervention	The LACI was significantly higher in patients who experienced MACEs when compared to those who did not.	[13]
Meloni et al., 2024	CMR	Patients with beta-thalassemia major	The LACI is significantly higher in beta-thalassemia major patients. The LACI is significantly higher in patients with beta-thalassemia after splenectomy compared to those with a spleen. An LACI >23.6% can be a good predictor of cardiac complications.	[40]
Huang et al., 2023	CMR	Patients with metabolic syndrome with and without metabolic dysfunction-associated fatty liver disease	The LACI is significantly higher in patients with metabolic syndrome. Metabolic dysfunction-associated fatty liver disease is an independent factor influencing the LACI.	[41]
Liu et al., 2024	CMR	Patients after myocardial infarction with and without metabolic syndrome	The LACI is significantly higher in patients after myocardial infarction. There is no significant correlation between the presence of metabolic syndrome and the LACI.	[42]
Wang et al., 2025	CMR	Patients with light-chain amyloidosis	The LACI is an independent all-cause mortality predictor in light-chain amyloidosis in patients with Mayo stages IIIa and IIIb. The LACI may be useful in risk assessment in advanced light-chain amyloidosis.	[43]
Yin et al., 2023	CMR	Volunteers with higher economic status	The LACI is significantly higher in patients with high urinary sodium. The LACI significantly correlates with BMI.	[44]
Pezel et al., 2022	CMR	Perimenopausal women without recognized cardiovascular diseases (MESA population)	The LACI is significantly higher in postmenopausal women than in premenopausal women. The LACI is significantly lower in perimenopausal women taking hormone therapy. The LACI is an independent predictor of atrial fibrillation, heart failure, coronary heart disease death and other hard cardiovascular disease.	[45]

## Data Availability

No new data were created or analyzed in this study. Data sharing is not applicable to this article.
